# A Hazard-Based Approach Enables the Efficient Identification of Chemicals of Concern in Plastics

**DOI:** 10.1021/acs.est.5c02912

**Published:** 2025-07-30

**Authors:** John D. Hader, Martin Wagner, Hans Peter H. Arp, Ksenia J. Groh, Mari Engvig Løseth, Laura Monclús, Jane Muncke, Lisa Zimmermann, Zhanyun Wang

**Affiliations:** † Empa—Swiss Federal Laboratories for Materials Science and Technology, St. Gallen 9014, Switzerland; ‡ 8018 Norwegian University of Science and Technology, Trondheim 7491, Norway; § Norwegian Geotechnical Institute, Oslo 0484, Norway; ∥ 28499 Eawag—Swiss Federal Institute of Aquatic Science and Technology, Dübendorf 8600, Switzerland; ⊥ Food Packaging Forum Foundation, Zürich 8045, Switzerland

**Keywords:** Global Plastics Treaty, hazard-based approach, regrettable substitutions, chemicals management, alternatives, hazardous chemicals

## Abstract

Plastics are composed of complex chemical mixtures, resulting in many chemicals being released during plastic’s life cycle, alongside a range of actual or potential impacts on human health and the environment. Many plastic chemicals also hinder technological solutions toward a safe and sustainable circular economy. Hence, there is broad agreement to address so-called plastic chemicals of concern, including under the Global Plastics Treaty. However, debate on how to identify such chemicals of concern is ongoing, particularly around whether their risk (and by extension, exposure) should be considered. In this perspective, we provide a review of the difficulties associated with understanding human and ecosystem exposure to and risks from plastic chemicals. Based on this, we highlight benefits of applying a hazard-based approach for identifying plastic chemicals of concern in a timely manner, and argue that additional consideration of exposure/risk would result in unjustified and costly delays, complications, and uncertainties, and therefore should not be required. A hazard-based approach to identifying plastic chemicals of concern would enable efficient action toward mitigating the impacts of plastics on human health and the environment, and facilitate a transition to a safe and sustainable plastics economy.

## Graphical abstract

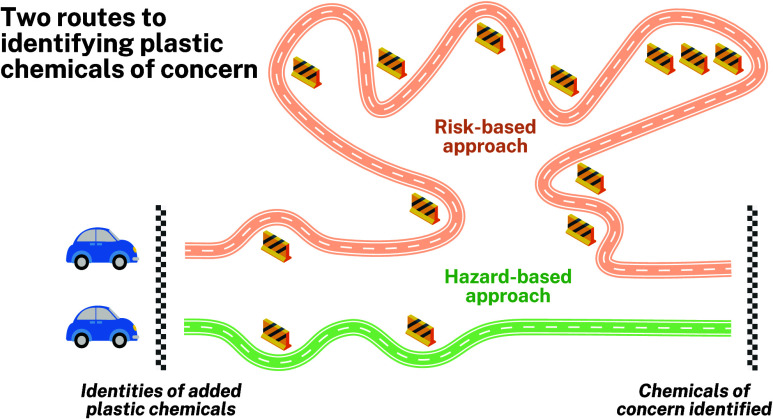

## 1 Plastic Chemicals

Plastics are key enablers of modern life. However, the growing amount of plastic produced, used, and disposed of by humanity pollutes and negatively impacts ecosystems and human health.^1–8^ In response, countries agreed in 2022 to work toward establishing an international, legally binding instrument on plastic pollution—a Global Plastics Treaty—to “prevent plastic pollution and its related risks to human health and adverse effects on human well-being and the environment,” covering the full life cycle of plastics.^9^ Five sessions of the Intergovernmental Negotiating Committing (INC) have occurred between 2022 and 2024, with the aim of finalizing and adopting the treaty text in 2025.^10^

Chemicals are an inherent component of plastics and form an important dimension of plastic pollution.^11,12^ Chemicals in plastics include starting substances (*e.g.*, monomers, catalysts), processing aids to enable or ease production and processing (*e.g.*, lubricants), and additives to maintain, enhance, and impart specific properties (*e.g.*, plasticizers, flame retardants, stabilizers).^13^ In addition to these intentionally added substances, non-intentionally added substances (NIAS) are present in plastics and include reaction byproducts, breakdown products, and impurities and contamination from the raw materials, production, processing, and recycling processes.^14–16^ Collectively, over 16,000 plastic chemicals have been identified for use, potential use, or presence in plastics. This encompasses approximately 13,000 plastic chemicals with known chemical structure information, and over 3000 that are of unknown composition.^17,18^ In accordance with previous work from several of the current authors, we define plastic chemicals as all chemicals that can be present in plastic products and materials, including the polymer backbone, intentionally added substances (*i.e.*, unreacted starting substances, additives, processing aids), and NIAS (*e.g.*, reaction byproducts, impurities, unreacted intermediates, and degradation products).^18^

Most plastic chemicals are not chemically bound to the polymer backbone, and the release of such plastic chemicals can occur at every stage of the life cycle—from production, processing, use, and recycling, to disposal or loss in the environment.^2,19,20^ Consequently, humans and the environment are continuously exposed to complex mixtures of plastic chemicals, which can result in adverse impacts on human and ecosystem health.^13,14,17,21–27^ Furthermore, the presence of chemicals in plastics can impede technological solutions addressing plastic pollution, including mechanical recycling, waste-to-energy, and chemical recycling.^12,17,28,29^

Accordingly, a broad consensus is emerging on the need to address chemicals of concern in the Global Plastics Treaty^30^ for achieving its aim of protecting human health and the environment.^9,12^ Currently, two competing approaches for identifying chemicals of concern have been discussed in the context of the Plastics Treaty,^31^ reflective of a long-running debate in the field of chemicals assessment and management.^32,33^ A “hazard-based” approach calls for chemicals to be identified as of concern based on their intrinsic hazardous properties, while a “risk-based” approach calls for additionally assessing the exposure of humans and wildlife to chemicals and determining the associated likelihood of adverse outcomes.

In this perspective, we elaborate why a hazard-based approach is not only scientifically robust and effective, but also the most efficient method for identifying plastic chemicals of concern. We present why requiring a risk-based approach would pose unjustified delays, complications, and uncertainties toward the sound management of plastics (Figure 1). Our rationale builds upon decades of lessons learned regarding exposure to anthropogenic chemicals—many of which are plastic chemicals—and the inherent challenges of exposure and risk assessment methodologies (Sections 2 and 3). Briefly, we argue that the large number of plastic chemicals, their variable composition within and across product categories, the lack of transparency regarding key information across supply chains, and the lack of chemical analytical capacity render infeasible a robust and efficient risk-based assessment of the plastic chemicals in commerce. These issues are compounded by the fact that providing strong evidence that links adverse outcomes to chemical exposures is inherently difficult and resource intensive. Finally, we provide recommendations on how to identify plastic chemicals of concern under the Global Plastics Treaty and other regulatory frameworks (building upon experiences from existing international treaties, *e.g.*, the Stockholm Convention on Persistent Organic Pollutants), call for transparency in the chemicals used in plastic production, and advocate for a simplification of plastic chemicals (Section 4).

**Figure 1:**
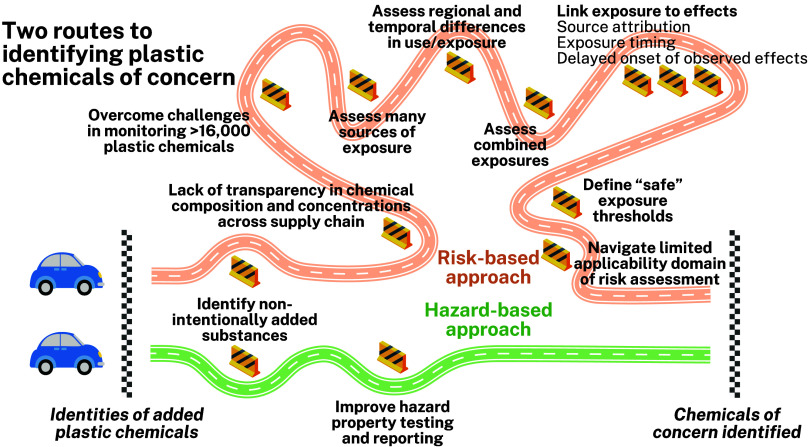
Key obstacles associated with identifying plastic chemicals of concern utilizing a risk- versus a hazard-based approach. Based on the discussion presented in Sections 2 and 3.

We emphasize that this perspective does not aim to diminish the important role of exposure science and risk assessments in chemicals management. Instead, we confine our scope to the *identification* of chemicals of concern in plastics, for which we argue exposure and risk assessment is not essential. Exposure and risk assessments would, however, play an important role in the subsequent management of identified chemicals of concern.

## 2 Widespread Exposure to and Impacts from Plastic Chemicals

Thousands of chemicals can be released from plastics and consequently contaminate water, air, soil, and packaged goods such as food.^34^ For example, at least 1210 plastic chemicals have been found to migrate from food packaging into food or food simulants.^23^ The full extent of chemicals leaching from plastics is probably larger, with recent studies demonstrating that a single plastic product may leach >2000 chemical features under realistic use conditions.^35,36^ Many plastic chemicals have been ubiquitously detected in the environment due to the global trade of plastic materials and the dispersal of plastic pollution.^37–39^ Plastic chemicals can be present in various environmental matrices, including in remote areas far from sources (*e.g.*, Polar regions), as shown for *ortho-*phthalates,^40^ organophosphate esters,^41^ bisphenols,^42,43^ polybrominated diphenyl ethers (PBDEs),^44^ and UV-328.^45^

To date, more than 1300 chemicals in plastic food packaging have been detected in humans,^46,47^ with studies detecting many *ortho-*phthalates,^48,49^ bisphenols,^50^ phenol antioxidants,^51^ and PBDEs^52^ widely in human populations, including at unsafe levels. Importantly, women, children, those with low incomes, and racial/ethnic minorities often have higher exposures to plastic chemicals than the wider population.^53–55^ The widespread environmental contamination by plastic chemicals also leads to wildlife exposures, as supported by a substantial body of literature. For example, Tyler et al. review how chronic exposure to several plastic chemicals (*i.e.*, PBDEs, BPA, and phthalates) can impact wildlife reproduction and development at environmentally relevant concentrations, Zhu et al. provide evidence for several plasticizers being present in Arctic biota, and Qadeer et al. compile information on the environmental occurrence, behavior, and possible effects of emerging and alternative plasticizers (*e.g.*, various adipates, phosphate esters, and trimellitates), while noting there is a lack of bioaccumulation data for many of these chemicals.^56–58^

Plastic chemicals may cause multifaceted adverse effects on human health and the environment. The potential for biological impacts at the individual and/or population scales is well-documented for some plastic chemicals, such as bisphenol A (BPA), some *ortho-*phthalates, flame retardants such as PBDEs, (short-chain) chlorinated paraffins, per- and polyfluoroalkyl substances (PFASs) used for fluoropolymer production, and cadmium and lead found in plastic utensils.^59–69^ Associations have also been documented between certain plastic chemicals and noncommunicable diseases, such as metabolic/endocrine, cardiovascular, and reproductive diseases.^62,70^ Apart from direct human or environmental harm, biocides in plastics can promote antimicrobial resistance,^71^ and plastic chemicals that act as greenhouse gases (such as hydrofluorocarbons used as blowing agents) can impact Earth systems function by contributing to climate change.^72^ Some adverse impacts from plastic chemicals are long-lasting and poorly reversible (*e.g.*, PFAS contamination, climate change),^72,73^ may be induced at very low concentrations (*e.g.*, endocrine disrupting chemicals mimicking hormone signaling),^74^ and/or may only be observed years after initial exposure (*e.g.*, prenatal exposure to endocrine disrupting chemicals and effects on childhood health).^75^

## 3 Challenges of Exposure and Risk Assessment for the Identification of Plastic Chemicals of Concern

Despite robust evidence for the ubiquitous release of plastic chemicals into built and natural environments, it is practically impossible to reliably quantify the full exposure to and associated risks posed to humans and wildlife from the thousands of plastic chemicals, even for some well-studied ones. Such comprehensive assessment is prevented by a lack of transparency in the use and supply chain of plastic chemicals, the chemical complexities of plastics, analytical challenges, difficulties in effect attribution, and overall limitations of exposure and risk assessment. While several of the issues associated with a risk-based approach (especially those pertaining to transparency and complexity) also apply to a hazard-based approach, in many instances these are much more difficult to overcome with a risk-based approach. We discuss these issues further in the following subsections.

### 3.1 Lack of Transparency

The lack of transparency regarding the chemical identity, production volume, uses, and levels of chemicals in individual plastic products hinders (independent) exposure assessments. It is reasonable to expect that industries possess data about the various chemicals they use in plastic production. While this information is critical for understanding associated releases and exposure, it is generally not made publicly available, including under the claim of confidential business information.^13,14^ Such information gaps exist not only in the public domain, but also along many supply chains, limiting the ability of businesses to take informed action (especially those further down the supply chain).

Even if some chemical exposure information is shared with and assessed by regulators in some jurisdictions, it is typically limited to the domestic situation and fails to consider the trade and development of chemical use over time. Thus, actual exposure levels can be much higher than initially anticipated during premarket approval.^49,76^ Furthermore, in contrast to intentionally added chemicals, knowledge on the types and levels of NIAS is mostly lacking.^16,24,77^

Current recycling practices further complicate exposure assessments, due to a general lack of tracing of recycled plastics and chemicals therein,^28^ and because recycled plastics may be utilized in applications with exposure characteristics different from their previous use. For example, recycled plastics from electronic products can end up in food contact applications and children’s toys.^63,78–80^

For the majority of plastic chemicals, hazard data are also missing. While several thousand plastic chemicals have been tested for their intrinsic hazardous properties (*e.g.*, related to their persistence, bioaccumulation, mobility, or toxicity), over 10,000 of the >16,000 chemicals used, potentially used, or detected in plastics lack hazard data in major regulatory hazard classification databases.^17^ Many plastic chemicals are therefore still being used while their health and environmental hazards are not known publicly^13^ or cannot be independently verified.

Due to the multifaceted lack of transparency, exposure assessments rely on the serendipity and technical capabilities of researchers to discover and quantify plastic chemicals, often requiring advanced analysis equipment (*e.g.*, nontargeted screening) and expensive monitoring campaigns. While transparency in chemical composition and hazards is crucial for any approach to identifying plastic chemicals of concern, a hazard-based approach avoids the complex issues of having to understand and quantify which chemical exposures occur from which product, at what levels, and at which stage of the product’s life cycle.

### 3.2 Chemical Complexity of Plastics

There are four layers of complexities associated with exposure and risk assessments of plastic chemicals: (i) the vast number of plastic chemicals, (ii) the diversity of their uses, (iii) regional and temporal variation, and (iv) legacy compounds from old products still in use or present in the environment.

First, the sheer number of >16,000 known plastic chemicals is challenging for exposure and risk assessment. This complexity is compounded by their diverse physical-chemical properties and their presence as mixtures, requiring a wide range of advanced analytical techniques to comprehensively assess the chemical composition of any given plastic sample.^81^ As discussed further in Section 4, this large number of plastic chemicals underscores the substantial workload needed for identifying chemicals of concern, regardless of the approach taken. This highlights the overall need for pragmatic and efficient strategies, noting that a hazard-based approach may help reduce the complexity of the task, as further elaborated below.

Second, even similar plastic items made of the same base polymer can have different chemical compositions,^35^ because manufacturers often use different plastic chemicals to impart the same function.^13,17^ Differences in the raw materials (*e.g.*, their purity) and production/recycling processes can also generate different NIAS, resulting in a multitude of chemical-product combinations that would have to be assessed, and different concentrations of the same chemicals in individual products.^82,83^ Thus, one would need to analyze a large and diverse set of plastic products for a large and diverse set of chemicals to obtain a representative overview of the chemical exposures from specific products.

Third, cultural practices, industrial activities, and regulatory frameworks differ across countries and change over time, resulting in diverse patterns of chemical use and exposure.^50,84^ Understanding and accounting for these regional and temporal variations is crucial for comprehensive assessments of exposure globally.^85^ Ever-intensifying global trade, including illegal trafficking of plastic waste, compounds this layer of complexity and impedes tracing the global movement of plastic products and waste, and associated exposures.^86–88^

Fourth, “legacy” chemicals present in old plastic still in use are relevant for exposures, as are persistent plastic chemicals previously emitted into the environment.^37,66^ For instance, PBDEs and PFASs previously used in and released from plastics remain in the environment for a long time due to their highly persistent nature, causing continued exposure, including in remote areas.^89–91^ Even nonpersistent chemicals may be preserved and transported *via* plastic debris and continuously released from the bulk plastic, causing long-term exposure.^92^

### 3.3 Analytical and Capacity Challenges

Despite recent advances in analytical methods for environmental contaminants,^38^ considerable challenges persist, impeding characterization of many plastic chemicals. Analytical standards for plastic chemicals are often missing, which are necessary to verify and quantify the compound under analysis.^93^ For example, Bradley and Coulier^77^ found that for six commodity plastic types—wherein the plastics were custom prepared and molded under laboratory conditions—many chemicals in extracts from the plastics remained unidentified. Similarly, Stevens et al.^94^ were unable to identify many features present in extracts from plastic food packaging purchased from domestic retailers (see also Zimmermann et al.).^36^ Additionally, plastics can contain >1000 substances of “unknown or variable composition, complex reaction products, or biological materials” (UVCBs).^17^ The chemical identities of these mixtures are poorly defined,^95^ hampering development of appropriate analytical methods.

Recent advancements in nontargeted analysis using high-resolution mass spectrometry can address some of the challenges to identifying unknown chemicals, but require advanced instrumentation, expertise, and time.^96^ However, low- and middle-income countries, and some high-income countries, often lack the capacity to conduct traditional targeted analytical measurements, let alone novel nontargeted analysis.^97,98^ Thus, empirical data for most plastic chemicals are scarce, making comprehensive exposure assessments unfeasible.^85^

### 3.4 Issues with Biomonitoring, Epidemiology, and Effect Attribution

If sufficient technical and financial capacity would be available to allow for a broad range of plastic chemicals to be biomonitored in humans and wildlife, and if observed adverse outcomes can be attributed to specific chemicals, this information can further be used to assess chemical risks and guide chemicals management strategies. However, relying on such a reactive strategy is problematic, as it faces many technical challenges, including obtaining ethical approval,^99^ while allowing exposure and potential adverse effects to continue.^100^ First, to detect chemicals in biota, exposure must have already occurred in a population at levels high enough to reach analytical quantification limits. At this point, adverse impacts may have already occurred,^101^ such as for chemicals for which early-life exposures are especially detrimental (*e.g.*, endocrine disrupting chemicals)^102,103^ and substances which can accumulate and persist in exposed biota.^104,105^ Second, linking such exposure to effects is challenging, since population impacts may take years to manifest after initial exposure.^75^ This temporal gap significantly limits the ability to establish causal links between exposures and effects, as the relevant exposure information is often lost at the time when adverse effects are observable.^106^ Additionally, the adverse effects must be large enough at the time of observation to be detectable.^107,108^

Linking effects to chemicals is further complicated by humans and wildlife being exposed to plastic chemicals as mixtures.^109^ Even when the effect-causing chemicals are identified, it can be difficult to identify their sources of exposure, exacerbated by the aforementioned lack of transparency around the chemical composition of plastics. Transformation products of chemicals, particularly metabolization products produced *in vivo*, pose additional challenges as their identity is often unknown and may be hard to trace from the degradation products identified.^110^ For example, it took over a decade to attribute the prevalent deaths of wild salmon near highways after rainfalls to 6PPD-quinone, an oxidation product of a common antioxidant used in tire rubber.^111^

### 3.5 Limitations of Exposure and Risk Assessment Methodologies

Nonbiomonitoring exposure assessments often employ exposure scenarios and/or computational modeling to (semi)­quantify exposures to chemicals.^112–114^ Currently, particularly when applied in a regulatory context, these assessments are typically used for a very narrow scope of use, such as food contact materials.^115^ However, to fully assess exposures, readily available data for plastic chemicals’ production, use, and end-of-life fate across assessed products would be needed. Such comprehensive data are generally lacking (see Subsection 3.1) and would typically only provide information for a snapshot in time. Without such transparency, as with epidemiology-based effect attribution, exposure assessments can only be done for substances already on the market and where analytical references are available, based on time- and resource-consuming monitoring data and uncertain assumptions to fill data gaps. Thus, once plastic chemicals are identified as being of concern based on an exposure assessment, it is often already too late to mitigate their adverse effects.

Furthermore, safety thresholds for chemicals are continuously refined—typically drastically reduced—as science and safety standards advance. For example, health-based drinking water guidelines for lead were 50 μg/L in 1984, whereas now there is not considered any safe level of lead exposure.^116–118^ The tolerable daily intake of BPA was similarly lowered from 4 μg/kg body weight per day in 2015 to 0.0002 μg/kg per day in 2023,^119^ classifying all people in Europe as being at significant risk.^120^ The reassessment of safety thresholds increases the precariousness of using risk assessment to identify chemicals of concern, as exposure levels previously considered safe may suddenly no longer be acceptable.^49^

The longevity of plastics used in many sectors—such as automobiles, construction, textiles, and electronics—represents another challenge to comprehensive understanding of exposures. Even when chemicals of concern in these products are identified, phasing out products in use and replacing them with products containing safer alternatives has typically been a slow process.^66^ Resistance to change due to lock-in^121^ and other hurdles, such as long innovation cycles and the need for testing and validation, contribute to a delayed transition away from hazardous substances. This can be seen by long exemption periods for decaBDE and Dechlorane Plus in spare automotive parts under the Stockholm Convention,^122,123^ resulting in continuing exposure to these chemicals while suitable alternatives are found and implemented.

As a result of the above challenges, exposure-dependent risk assessments are complex, highly uncertain, resource-intensive, and not suitable for the timely identification of plastic chemicals of concern. They would cause long, avoidable, and unjustified delays, potentially resulting in substantial societal costs associated with adverse effects on human health and the environment, and impairment of a transition to a safe and sustainable economy.

## 4 Recommendations on the Way Forward

Based on the discussion above, we provide the following three recommendations for addressing plastic chemicals of concern, including under the future Global Plastics Treaty.

### 4.1 Use a Hazard-Based Approach to Identify Plastic Chemicals of Concern

The approach under the Stockholm Convention on Persistent Organic Pollutants lends a helpful model for identifying plastic chemicals of concern. In brief, the Convention identifies chemicals that warrant global action based on predefined hazard criteria (*i.e.*, persistence, bioaccumulation, long-range transport potential, and toxicity), accompanied by qualitative considerations related to exposure (*e.g.*, evidence of exposure in local areas, especially due to long-range transport). In a subsequent step, consideration of specific uses of a chemical informs whether any action requires specific exemptions within a risk management context.^124^

For plastic chemicals, a similar two-step approach could be implemented. In a first step, plastic chemicals of concern may be identified based on predefined, scientifically robust hazard criteria. While discussing which hazard criteria should be used is outside the scope of this article, studies and methods exist employing a hazard-based approach for screening plastic chemicals. For example, Wagner et al. employed an approach of using persistence, mobility, bioaccumulation, and/or toxicity as hazard criteria to identify plastic chemicals of concern, and subsequently prioritized these chemicals for action based on their hazardous properties (or lack of hazard data), regulatory status, production volumes, and data on the chemicals’ detection in and migration from plastics (see also Monclús et al.).^17,18^ When employing these four criteria for a hazard-based approach to identifying plastic chemicals of concern, >4200 of the >16,000 plastic chemicals are identified as being of concern. Zimmermann et al. similarly identified food contact chemicals of concern using hazard properties of concern defined by the EU’s Chemicals Strategy for Sustainability (*i.e.*, carcinogenic, mutagenic, toxic to reproduction, specific organ toxicity, persistent and bioaccumulative, persistent and mobile, or endocrine disrupting).^27^ Additionally, further hazard criteria, including mobility of chemicals in media other than air and water, may be considered. The selection of specific criteria warrants a separate and more in-depth analysis. Regardless of the specific hazard criteria selected, the agreed criteria should be flexible enough to include new hazard classes as science progresses. These hazard criteria could be complemented (if needed) by qualitative exposure considerations, such as whether there is evidence for the use or detection of the assessed chemical in plastics, or whether its presence is plausible. In a second step, risk management decisions would be made on the required action to address such identified plastic chemicals of concern based on what can be understood regarding their uses, releases, and exposure, and the need for any exemptions.

To enable these two steps, we propose a workflow for soundly managing plastic chemicals (Figure 2). First, **identification of chemicals of concern** occurs using predefined hazard criteria. For such chemicals of concern, an **essentiality assessment** then determines whether the chemical provides a function essential to the overall functionality of the plastic product (particularly in the context of health, safety, or functioning of society), and if so, whether there are alternative chemicals not of concern that could be used to supply this function instead.^125,126^ **Functionally unnecessary** plastic chemicals of concern can be phased out quickly.^127^ Through **alternatives assessment**, functionally necessary chemicals may be replaced with chemicals that are not of concern and supply the same essential functionality to the plastic product in a timely manner.^125,128^ Note that identification of chemicals of concern and safe alternatives is limited by available hazard information; see Section 3 and subsection 4.2 below. For chemicals in uses deemed essential and where no safe alternative exists, **exposure and risk assessments** can be introduced to highlight hot and blind spots of exposure and risk and inform further exposure reduction measures. Plastic chemicals that are of concern, are deemed essential, and have undergone exposure and risk assessment may furthermore be prioritized for **concerted action to remove/replace** them in the market.^126^ Ultimately, in-depth exposure and risk assessment should be seen as a last line of defense for the safer use of plastic chemicals, with hazard assessment being the first. Furthermore, the hazard-based approach to identifying plastic chemicals of concern aligns with the overarching Safe and Sustainable by Design (SSbD) approach, which emphasizes avoiding hazardous chemicals in products at the design phase. Consequently, SSbD offers a solution framework for managing chemicals of concern by promoting the substitution of hazardous chemicals in products, with the ultimate goal of making such products safe for use across their known and unforeseen life cycles.^129^

**Figure 2:**
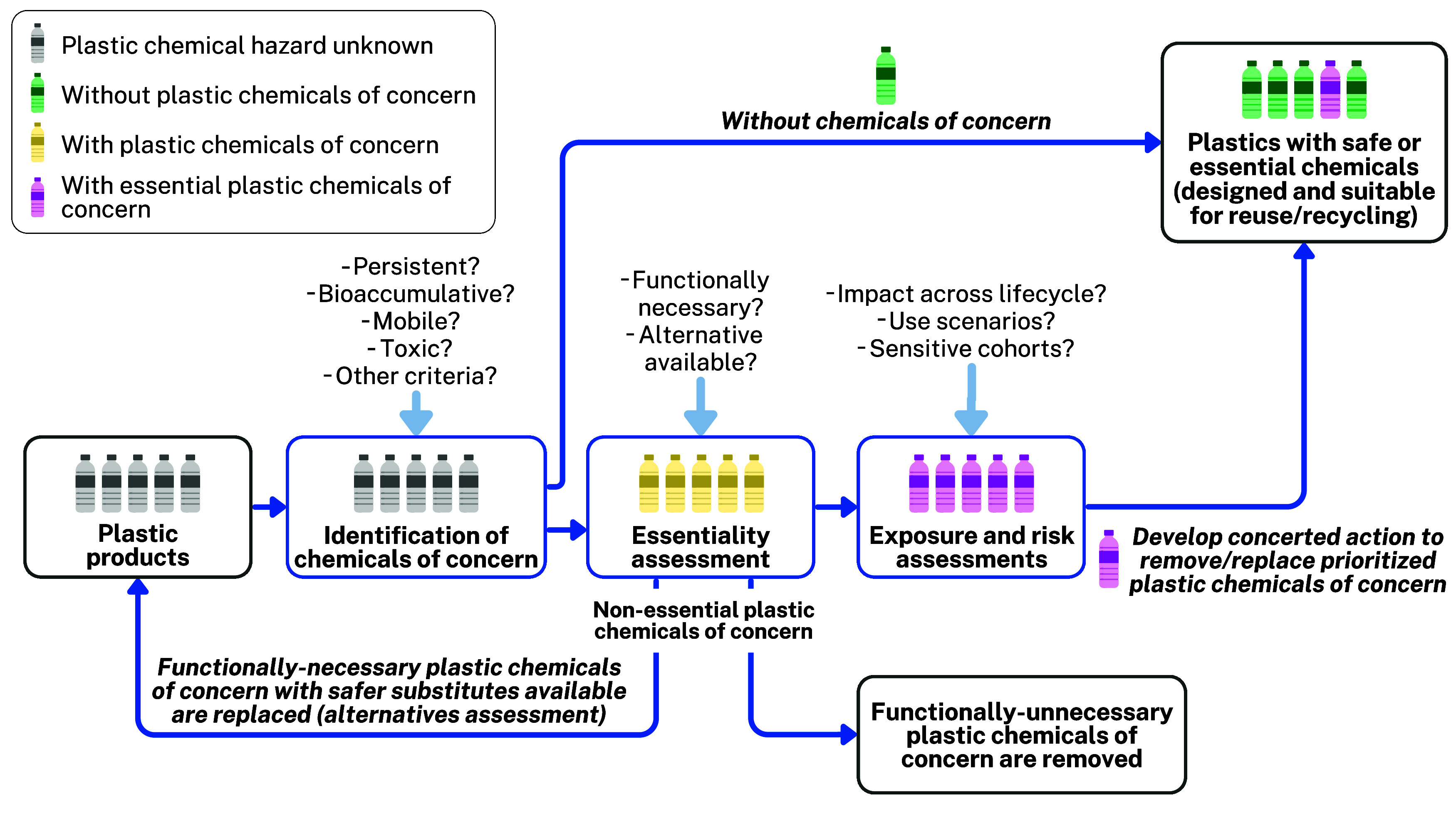
Proposed workflow for soundly managing plastic chemicals, following the hierarchy of a hazard-based approach to identify chemicals of concern, assessment of essentiality of chemicals of concern, and exposure and risk assessments, with the latter two steps guiding the development of effective risk management measures.

### 4.2 Enhance Transparency and Traceability of Plastic Chemicals

To soundly manage plastic chemicals, society must ensure transparency of the chemical composition of plastics, regarding both the identities and levels of compounds in specific applications. To do so, a public database listing all intentionally added chemicals per plastic product being placed on the market may be implemented by industry. For example, the global automobile industry has developed an internal International Material Data System, where “all materials used for automobile manufacturing are collected, maintained, analysed and archived.”^130^ Such efforts could be copied by other sectors and made publicly accessible. As another example, the International Council of Chemical Associations (ICCA) is hosting a Plastic Additives Database with information on chemical additive function, the associated polymer type, and the industrial sector using the additives.^131,132^ Methods for tracing chemicals present in plastics along their supply chain and entire life cycle are also under development, such as part of the EU’s Ecodesign for Sustainable Products Regulation and the associated Digital Product Passport initiative.^133^ This matter may merit more detailed analysis in the near future. Data availability could also be a regulatory requirement, as is the case with the EU’s “Substances of Concern In articles as such or in complex objects (Products),” or SCIP, Database.^134^

For NIAS—the majority of which have unknown chemical identities—plastic production processes should be optimized and standardized to limit their variety and levels in plastics. To accomplish this, additional mechanistic understanding of NIAS sources or formation processes, and enhanced capabilities for NIAS identification, are needed.^12,77,94^ This could be assisted by making more repositories of NIAS and their introduction pathways available on open databases, such as the Norman Substance List Exchange,^135^ as has been proposed by others.^136^ Transparency around the identities of UVCBs has also been identified as an issue previously. Acknowledging the complexity of this issue—as well as recent advancements in analytical and data technologies—several potential solutions may be considered. These may include making commercial samples available for broad public analytical testing, making producer and/or regulatory data on these compounds more publicly available, aggregating existing UVCB data into a more centralized hub, and additional development and adoption of novel, standardized chemical structure identifiers.^95^ Expanding suspect and nontargeted analytical capacity would also support monitoring and identification of plastic chemicals in the built and natural environment, where plastics with high production volumes and/or exposure potential (*e.g.*, food contact materials) should be prioritized.^12,70^ Here, a global mechanism, such as a Global Plastics Treaty, can promote or mandate creation of global networks to identify, monitor, and prioritize plastic chemicals.^12^

Furthermore, for most plastic chemicals, the lack of hazard information poses a serious challenge to the identification of chemicals of concern—both for the hazard- and risk-based approaches. New methods for assessing such hazards, enhanced data collection and reporting outlets, and technical capacity building are thus urgently needed.^17,128^

### 4.3 Encourage Simplified, Standardized, and Safe Plastic Chemicals

In parallel with managing chemicals of concern in existing plastics, concerted efforts should be made to develop a new generation of safer plastics and alternative materials, in which chemicals allowed in their production are simplified, standardized, and rigorously checked for safety. Plastic formulations should also be harmonized toward more reusability and recyclability.^12,137,138^ Technical solutions to plastic pollution are hindered by the current approach to plastic chemicals management, as some plastic chemicals can cause adverse effects on technical systems. For example, many plastic chemicals reduce the marketability of mechanically recycled plastics, resulting in downcycling or waste.^12^ The presence of halogenated substances and metals in plastics also increases the cost and technical complexity of waste-to-energy and chemical recycling processes,^139,140^ posing technical challenges to utilizing these technologies.^12^

To implement a simplified, standardized, and safe approach to management of plastic chemicals, coordination will be needed from industry and other stakeholders across the whole value chain to agree on standards. Herein, international instruments—such as a Global Plastics Treaty—can help to direct funding to create a level playing field.^12,21^ Standardization would not be a hindrance to innovation, but rather a context for it, wherein innovation would shift finite resources toward optimizing the safety, transparency, logistics, and profitability of plastics, crucial for transitioning to a more sustainable and circular (plastics) economy.

The problem-solving principle of Ockham’s Razor is that additional complexity should only be introduced when necessary to solve a problem. While the universe of chemicals used in plastic products is a complex space, the hazard-based approach to identifying which of these chemicals are of concern offers an efficient, simple, and fit-for-purpose solution to take a timely first step toward enabling a safer use of plastic chemicals. More complex exposure and risk assessments should be reserved only for subsequent steps to develop concerted action on plastic chemicals of concern once they are identified.
